# The Power of Pediatric Sleep: Shaping Healthy Evidence‐Based 24/7 Lifestyles for Generation Alpha

**DOI:** 10.1002/pdi3.70018

**Published:** 2025-08-21

**Authors:** Karen Spruyt

**Affiliations:** ^1^ INSERM Université Paris Cité Paris France

**Keywords:** child development, evidence‐based sleep practices, pediatric sleep

Pediatric sleep is a crucial but often overlooked component of child health, influencing development, well‐being, and long‐term outcomes. As our understanding of pediatric sleep deepens, the need for evidence‐based approaches to pediatric sleep practices becomes increasingly vital. This editorial seeks to highlight the importance of evidence‐based pediatric sleep practices and its integration into holistic evidence‐based lifestyle management that promote optimal child health.

Historically, sleep has been undervalued in healthcare despite being foundational for growth, cognitive development, and energy regulation. During the early weeks of life, the sleep state is disrupted as wakefulness gradually becomes more dominant, transitioning into a 24 h cycle of sleep, feeding, and alertness (e.g., the first concepts of this were introduced in 1937 by Kleitman). As activities, such as play, learning, and eating, take precedence, the importance of sleep in early childhood for emotional regulation, attachment, and security becomes even more evident. These early years of sleep are irreplaceable for cognitive and physical development, emphasizing the need for a shift toward prioritizing sleep as a cornerstone of pediatric care.

Generation Alpha, growing up amidst rapid technological advancements, will naturally embrace evidence‐based lifestyles supported by AI tools, wearables, and data monitoring (e.g., Ref. [1]). Devices, such as sleep trackers and AI‐powered diagnostics, are gradually transforming how pediatric sleep is personalized and optimized (e.g., Ref. [2, 3, 4, 5]). These technologies enable real‐time monitoring, providing data‐driven insights that allow for tailored interventions. Generation Alpha will use scientific data to optimize their health behaviors, including sleep, across a 24 h cycle.

This shift toward continuous data collection and the growing demand for reliable, evidence‐based health practices (e.g., Ref. [6]) require pediatric sleep research and practice to be rooted in robust, evidence‐driven methodologies. To advance this field, solid evidence, replication studies, and cross‐cultural data sharing are essential to ensure the validity and generalizability of findings. Advancements in pediatric sleep further require a transdisciplinary approach across health domains and developmental stages, grounded in developmental theory and guided by the needs and capabilities of families and pediatric sleep professionals, with support from industry partners to translate innovations into market‐ready solutions. Below, I outline key areas that should be prioritized to advance evidence‐based pediatric sleep practices.

## 1 Expanding Pediatric Sleep Research *Evidences*

Research highlights the critical role sleep plays in various aspects of child development, including cognitive function, emotional regulation, immune health, and physical growth (e.g., Ref. [7, 8, 9]). Sleep is not just a passive state but an active and dynamic process that consolidates learning, supports brain development, and stabilizes emotions. This emphasizes the need for prioritizing pediatric sleep health, especially during the early years of life.

Despite its importance, there are significant gaps in our understanding of pediatric sleep, including how sleep patterns develop, what constitutes healthy sleep at various developmental stages, and how cultural factors influence sleep behaviors.

Most data on pediatric sleep issues are derived from behavioral assessments, often using clinical cross‐sectional convenience samples, with a notable absence of standardized sleep questionnaires [10]. Sleep complaints are prevalent in typically developing children, affecting around 60% [11], and are even more prevalent in children with neurodevelopmental disorders [12, 13], with rates exceeding 90%. These children face a higher risk of issues such as difficulties of initiating sleep, frequent nighttime awakenings, sleep‐related breathing problems, reduced total sleep duration, and poor sleep quality. Sleep disorders, such as insomnia, affect 40%–80% of individuals with neurodevelopmental disabilities [14], with 50%–83% of children with autism spectrum disorder (ASD) experiencing insomnia and circadian rhythm sleep‐wake disorders [15]. In children with attention deficit hyperactivity disorder (ADHD), high rates of motor restlessness (50%) and parasomnias, such as sleepwalking (47.6%), night terrors (38%), and confusional arousals (28.5%), are reported. Obstructive sleep apnea is found in 50%–80% of children with Down syndrome [16] and around 80% of those with Prader–Willi syndrome [17], whereas up to 20% of typically developing preschoolers experience obstructive sleep apnea [18]. Restless leg syndrome symptoms are observed in 11%–43% of children with neurodevelopmental disabilities [19].

Sleep disturbances in children are often compounded by underlying pediatric conditions, complicating both diagnosis and therapeutic management. In cases of cancer, epilepsy, brain tumors, migraines, or rare diseases, multifactorial etiologies contribute to sleep impairments, though disentangling individual causal factors remains challenging—a barrier to developing targeted interventions [20, 21, 22, 23]. Similarly, for instance, type 1 diabetes is associated with disrupted sleep due to glycemic instability (which directly affects sleep architecture), nocturnal hypoglycemia management, and the demands of continuous glucose monitoring and insulin administration. Notably, sleep disruptions exhibit strong associations with childhood conditions, underscoring the importance of early detection and individualized treatment strategies.

However, the existing literature on this topic consists largely of small‐scale single‐center observational studies, which utilized diverse methodologies for measuring sleep and yielded conflicting results. Children and adolescents—particularly those with neurodevelopmental disorders—commonly experience diverse sleep disturbances that often cooccur with other sleep pathologies, medical conditions, psychiatric comorbidities, or medication effects. A persistent clinical challenge involves obtaining objective sleep measurements while reducing patient burden associated with standard polysomnographic monitoring in hospital settings. Population‐based studies highlight the lasting effects of poor sleep on neurocognitive and socioemotional development, emphasizing the need for early interventions to improve long‐term developmental outcomes (e.g., Ref. [24]).

Several studies have sought to identify key sleep trajectories across various parameters, including sleep duration, sleep problems, such as insomnia, night waking, and sleep onset difficulties, and bedsharing behaviors [25]. Their findings reveal considerable global diversity in sleep research, with notable gaps, particularly in low‐income regions and among neurodiverse populations. Most studies have concentrated on sleep trajectories in early childhood, with fewer focusing on school‐age children and adolescents. Although these studies offer valuable insights, they also highlight several caveats, such as inconsistent statistical quality, a lack of standardized tools, and the need for more prospective research, to improve understanding of childhood sleep trajectories and guide targeted interventions. The reliance on parental reports and limited self‐reports, along with variability in the determinants and outcomes studied, further complicates interpretation.

Although sleep duration is the most frequently studied parameter, few studies investigate it alongside other sleep problems. Despite abundant evidence linking poor sleep to negative behavioral and health outcomes [7]—primarily in clinical populations—defining and categorizing sleep trajectories remains challenging. Notably, however, their findings suggest that children may not always outgrow sleep problems, potentially indicating underlying conditions and long‐term risks. This underscores the importance of heightened awareness among healthcare professionals and the need to prioritize sleep health throughout childhood development.

Advancing pediatric sleep practices requires a rigorous evidence‐based approach with replication studies and aggregated data collection to validate findings (e.g., Ref. [26]). For instance, several studies highlight significant cultural differences in sleep patterns, including variations in duration and disturbances, across different regions [27, 28, 29]. Cultural factors, such as values, beliefs, and practices, shape sleep behaviors, with variations in bedtime routines, sleep arrangements, and physical and psychological factors influencing sleep outcomes. Additionally, factors that influence sleep in one culture may not be relevant in another, making it essential to consider the cultural context when applying tools, cutoffs, or interventions. Caution is warranted to ensure that sleep assessments and interventions are culturally appropriate.

Specifically, childhood sleep ecology differs from adult sleep patterns, requiring tailored assessment and intervention strategies that address developmental needs. For example, cognitive behavioral therapy for insomnia (CBTi) has demonstrated small but significant clinical effects in children and adolescents [30], or age‐appropriate approaches are needed to address issues such as excessive daytime sleepiness [31]. Overall, intervention research faces significant gaps and barriers. Most intervention studies focus on young children, with limited research on school‐age children and adolescents. Additionally, most participants come from WEIRD (Western, Educated, Industrialized, Rich, and Democratic) societies, raising concerns about the cultural relevance and effectiveness of proposed behavioral treatments in the literature. Moreover, there is a lack of randomized controlled trials, along with significant variability in aspects, such as treatment delivery, age ranges, family involvement, outcomes, and (long‐term) follow‐up, among intervention studies. Yet, common nonpharmacological interventions, including bedtime routines, parent education, sleep hygiene, and graduated extinction, are typically the first‐line treatment options.

Research on pharmacotherapy for pediatric sleep disorders is limited, with medications often prescribed off‐label based on adult studies. Given the absence of U.S. Food and Drug Administration (FDA), European Medicines Agency (EMA), and Chinese National Medical Products Administration (NMPA) approved medications for pediatric sleep disorders, except for narcolepsy, clinicians must be well‐versed in sleep disorder classifications and the appropriate role of pharmacotherapy when considering treatment options. A thorough risk‐benefit analysis is essential, and clinicians should involve families in the decision‐making process. For children with developmental disabilities, medication selection and dosing should be individualized, considering the severity and nature of the sleep disorder as well as any cooccurring neurodevelopmental or medical conditions. Due to a lack of sufficient robust, clinical, and evidence‐based data, there is ongoing uncertainty regarding optimal dosing and tolerability.

Melatonin is a notable exception. Melatonin is commonly used to treat insomnia in children and adolescents, but clinical guidelines for its use are limited due to insufficient or inconclusive data. In response, the International Pediatric Sleep Association (IPSA) has developed expert recommendations for its use in pediatric insomnia. For typically developing children, there is limited empirical evidence supporting its safety and efficacy in treating chronic insomnia [32]. Given that, melatonin is available over‐the‐counter in countries, such as the U.S., Canada, and China, and the prevalence of conflicting online advice, caregivers, and clinicians need accurate guidance to make informed decisions. For children with neurodevelopmental disorders, including autism [33], melatonin is often prescribed for sleep difficulties. However, its use requires careful consideration of potential side effects, given its hormonal nature [34]. Although melatonin can be effective for short‐term sleep issues, it should be used under the supervision of a healthcare provider as part of a comprehensive treatment plan that includes nonpharmacological approaches. Factors, such as dosage, melatonin composition, timing, duration, the child's endogenous melatonin levels, and the form of administration (e.g., gummies, pills, or soluble), must be tailored to the individual child's needs and administered under supervision. To minimize the risk of overdose or long‐term use [32, 35], dosage should be carefully discussed and melatonin levels assessed through methods such as dim light melatonin onset [36] or morning voids [37]. Although melatonin is generally well tolerated, children may experience side effects, with their severity, frequency, and long‐term impact still remaining uncertain [38]. Only through rigorous research‐driven efforts can we ensure that pediatric sleep interventions are based on reliable scientifically backed insights rather than anecdotal evidence or trends.

As awareness of pediatric sleep disorders grows, researches into their diagnosis, treatment, and prevention are rapidly expanding. However, also a significant gap persists between research findings and their translation into clinical practice [39] and in shaping parenting attitudes. Bridging this divide necessitates a stronger focus on evidence implementation to enhance real‐world pediatric health care.

## 2 Enhancing Clinical Expertise *Evidences*

A major barrier to improving pediatric sleep practices is the limited availability of specialized training for healthcare providers. Although some international sleep societies have started offering pediatric sleep training, there is a pressing need for more hands‐on education and collaborative sharing among clinicians [39]. Healthcare providers need both foundational and advanced expertise in managing common pediatric sleep disorders, such as obstructive sleep apnea, insomnia, and narcolepsy [39]. Furthermore, healthcare providers across clinical settings must maintain expertise in identifying and addressing sleep disturbances in children with neurodevelopmental disorders (e.g., ASD, ADHD, and Down syndrome) and other pediatric conditions. For example, sleep disorders represent the most common comorbidity in pediatric migraine patients, demonstrating significantly elevated prevalence rates of parasomnias, obstructive sleep apnea, and sleep‐related movement disorders versus unaffected children.

Particularly, vulnerable are critically ill pediatric patients in intensive care settings, where multifactorial risks—including physical stressors, environmental disruptions, and pharmacologic interventions—collectively impair sleep quality. These sleep disturbances may negatively influence both acute recovery trajectories and long‐term neurodevelopmental outcomes. The complex sleep issues observed in these populations, ranging from insomnia to circadian rhythm dysregulation, demand patient‐specific diagnostic and therapeutic approaches. Qualifications and a standardized certification process are essential to ensure competency‐based interprofessional training, especially with the growing challenge posed by “AI consultants”. Currently, the demand for clinical pediatric sleep care surpasses workforce capacity, presenting both challenges and opportunities to train a diverse skilled team.

Sleep is shaped by biological, cultural, and societal factors, meaning what is considered “normal” sleep varies across cultures. Sleep behaviors, influenced by the family context, are shaped by cultural norms and societal factors such as parental leave, shift work, and daycare policies. These factors contribute to both commonalities and differences across cultures in parenting practices and child outcomes. A clear example of societal norms, beliefs, and policies's effect is the role of school start times on children's sleep habits. Thus, integrating cross‐cultural data sharing and collaborative research are crucial to advancing pediatric sleep practices, including our clinical perspectives. Comparative studies across cultures can reveal both universal sleep patterns and societal specific variations, helping to create globally relevant and culturally sensitive guidelines. These approaches ensure that sleep interventions are tailored to the diverse needs of children, promoting equitable care. However, funding limitations and logistical challenges in cross‐cultural sleep research can hinder progress, underscoring the need for sustainable financial support and effective coordination. Collecting robust evidence will bridge the gap between clinical needs and practice, facilitating continuous professional development in an increasingly diverse and inclusive healthcare landscape.

In addition to being underfunded as a multidisciplinary field, progress in sleep research is hindered by factors such as the high cost and limited access to gold‐standard sleep recording methods (e.g., in‐hospital polysomnography), methodological variability, low‐quality evidence, a lack of longitudinal data, overreliance on convenience (clinical) sampling, and a shortage of replication studies. Therefore, technological innovations, including wearables, sleep trackers, and AI‐driven diagnostics, offer opportunities to refine pediatric sleep practices, fueled by growing industry interest and funding. These technologies enhance the accuracy of diagnostics, enable real‐time monitoring, and allow for individualized treatment plans based on real data. Although technological innovations offer significant potential for improving pediatric sleep practices, their potential risks must be considered. These risks include conflicts of interest, limitations, and ethical concerns, such as privacy issues related to wearables and data monitoring, as well as the danger of prioritizing technology over behavioral and environmental interventions. Additionally, the overreliance on technology may contribute to the development of technology‐induced sleep disorders, further complicating sleep management.

As the adage goes, “it takes a village to raise a child” (African proverb). Our research, for instance, highlights that children’s sleep is closely tied to the opportunities they have for sleep [40] and is influenced by family stressors [41]. This underscores that contextual variables must be carefully accounted for in both the application and interpretation of sleep monitoring devices or sleep trackers. This, in turn, suggests the need for family‐based child‐centered devices, cloud data sharing, and algorithms, fostering high‐tech interoperability. Such approaches require careful consideration of ethical issues, including privacy, compliance with the General Data Protection Regulation, data security risks, and challenges related to multicloud management, cybersecurity gaps, and potential human error. Ongoing initiatives, such as the European Open Science Cloud, aim to address these concerns and advance personalized health assistance and interaction support platforms. Ultimately, empowering youth to manage and regulate their 24 h cycle will depend on balancing consumer‐driven, community‐based, and evidence‐based scientific approaches, alongside the clinical expertise.

The integration of innovative technologies with evidence‐based clinical guidelines will ensure that interventions are both personalized and grounded in solid scientific research. A continuous around‐the‐clock sleep strategy that prioritizes enhancing sleep—rather than merely boosting wakeful brain activity (e.g., cognitive performance)—can greatly enhance pediatric health management, shaping the future of generations. This perspective prioritizes not only sleep quantity but also its quality, ensuring alignment with the natural circadian rhythm and developmental needs of children, as for example, demonstrated by research on school start times [42]. By fostering an environment that supports restorative sleep throughout the 24 h cycle, this approach enables better sleep outcomes, enhanced cognitive function, and emotional regulation, all of which are critical for healthy growth and development. Incorporating these technologies into everyday life enables real‐time data collection and provides precise tailored interventions that are both effective and sustainable. This approach aligns with the growing demand for evidence‐based practices that are adaptable to individual needs and family contexts, supporting sustainable lifelong health trajectories.

## 3 Integrating Patient Preferences *Evidences*

As the field of pediatric sleep evolves, there is a growing emphasis on personalized precision‐based care. This shift reflects not only the increasing awareness among healthcare providers but also the active involvement of parents in managing their children's sleep health. Parents are increasingly seeking individualized participatory approaches that cater to their child's unique sleep needs and circumstances.

For instance, children with neurodevelopmental disorders—including ASD, ADHD, Down syndrome, and rare genetic conditions—frequently experience profound sleep disturbances, such as insomnia, frequent nighttime awakenings, and circadian rhythm dysregulation. These patients and families often necessitate tailored individualized interventions to address their unique sleep issues. Similarly, sleep disturbances in pediatric patients with medical conditions may arise from primary sleep disorders, comorbidities with acute/chronic illnesses, or secondary effects of disease processes, treatments, or hospitalization. Effective clinical management requires a multidisciplinary approach that prioritizes patient‐centered and family‐inclusive care.

Emerging research highlights significant differences in sleep patterns between clinical settings. Notably, outpatient populations often demonstrate greater challenges with sleep initiation and early morning waking compared to hospitalized children. These findings underscore the critical need for participatory approaches that incorporate patient and family perspectives in both assessment and intervention strategies. However, despite the theoretical benefits of evidence‐based practices, implementing these methodologies at the patient level remains a challenge (e.g., Ref. [43, 44]) in clinical (research and) practice. Barriers include high attrition rates (up to 30%), the developmental nature of sleep‐related issues, the complexity of sleep disorders, variations in lifestyle/home environment, daytime demands on parents, and family relationship challenges, among others. Additionally, parents often struggle with finding qualified sleep experts, navigating complex treatment plans, and accessing appropriate care.

A 24/7 approach to pediatric sleep management is necessary to tailor sleep assessments and care to each child's specific needs. For example, as stated, melatonin—a hormone critical for regulating sleep‐wake cycles—is increasingly used in managing pediatric sleep disorders, particularly in children with neurodevelopmental conditions such as ASD and ADHD [32, 33, 38]. Although its short‐term efficacy in reducing sleep onset latency and enhancing sleep duration is frequently cited, the evidence remains limited and poorly established, warranting cautious consideration [32, 33, 38]. Concerns about long‐term safety, appropriate dosing, timing, and potential effects on the developing body and brain further emphasize the need for judicious evidence‐based use.

Parental preferences often lean toward noninvasive behavioral interventions, reflecting a desire for accessible sustainable solutions to their children's sleep challenges. Optimal treatment plans should seamlessly incorporate an assessment of medication usage, when appropriate, alongside behavioral and environmental interventions [45, 46]. Children with neurodevelopmental conditions often require tailored sleep interventions; for instance, those with ADHD may benefit from targeted sleep hygiene protocols addressing hyperactivity and restless behaviors characteristic of their condition. Meanwhile, research reveals hospitalized pediatric patients face compounded sleep challenges—studies demonstrate they frequently obtain insufficient sleep (both in duration and quality) during crucial recovery periods, often falling below recommended clinical thresholds. Environmental disruptors, such as excessive light and noise, are modifiable factors significantly impairing sleep continuity in hospital settings. These findings highlight the necessity for multidisciplinary collaboration among clinicians, administrators, and hospital designers to create restorative sleep environments. Critically, families prefer noninvasive solutions, including both environmental modifications (e.g., noise reduction and lighting controls) and behavioral strategies (e.g., sleep hygiene). An integrated approach ensures that caregivers and youth receive clear guidance and education.

To advance pediatric sleep practices, incorporating UNICEF’s (2017) parenting program standards could be valuable, particularly Standard 6, which emphasizes adapting interventions to cultural contexts, and Standard 7, which advocates for integrating programs into existing platforms. Such an approach would encourage positive parenting practices related to sleep, framed within societal norms and beliefs (e.g., Ref. [47, 48]). The increasing awareness, health literacy, and preferences of caregivers play a significant role in advancing pediatric sleep interventions and technology.

The practical challenges faced by parents or caregivers—such as identifying sleep experts, accessing them, and integrating them into their child's care plan—highlight the need for clear accessible resources. Providing comprehensive support for caregivers is critical to bridging the gap between research evidence and real‐world patient outcomes. By prioritizing caregiver education and involvement, pediatric sleep care can become more effective, inclusive, and aligned with the unique needs of each family.

## 4 The Impact of Evidence‐Based 24/7 Lifestyles

As Generation Alpha embraces evidence‐based lifestyles driven by data and technology, pediatric sleep medicine must keep pace with these developments. According to the U.S. Common Sense Census, data from 2020 demonstrate that young children spent an average of 39 min daily watching online videos, a substantial increase from 19 min in 2017. This rise is largely attributed to the increased access to mobile devices, with nearly half of 2‐ to 4‐year‐old children and over two‐thirds of 5‐ to 8‐year‐old children owning their own tablets or smartphones. However, the digital divide has widened, with socioeconomic and racial disparities becoming more pronounced [49]. Although mobile media use continues to grow, efforts to close the digital divide have stagnated, leaving many U.S. lower‐income families without reliable internet or access to devices. As social media usage increases among tweens and teens [49], the role of emerging media, including virtual reality, will significantly impact children's digital experiences. Much of the content children consume online is dominated by videos, often including advertisements rather than truly educational material. This exposure enables children to engage with various cultures, languages, and customs from a young age, fostering multicultural identities and promoting inclusivity and empathy. The challenge remains to ensure equitable access and meaningful engagement with technology for all families. Though approximately 90 apps (up to 2022) are available on platforms, such as Apple iTunes and Google Play, to improve sleep, few incorporate evidence‐based behavioral strategies [50, 51]. Although research on Generation Alpha's adoption of data‐driven evidence‐based lifestyles is still developing, further studies on its application to sleep health are needed.

The widespread access to technology, multicultural education, and cross‐cultural interactions in Generation Alpha suggests that they may be better positioned to tackle health challenges. As such, precision‐based sleep care and participatory sleep research are critical for fostering sustainable lifestyle changes grounded in solid scientific evidence. Ultimately, improving sleep is fundamental to shaping a healthier future.

Evidence‐based lifestyles enable individuals to integrate the latest research findings into daily routines, *prioritizing sleep*, to enhance quality of life and reducing the risk of chronic conditions (see Ref. [52]). Evidence‐based sleep practices may help restore a balanced wake‐sleep cycle, supporting a healthy 24 h rhythm of sleep, feeding, and alertness (e.g., play, learning, and interaction). For evidence‐based pediatric sleep practices to succeed, they must be personalized, sustainable, and culturally sensitive, addressing both individual and community health needs. This approach has the potential to significantly improve public health, with pediatric sleep practices playing a critical role in optimizing child health and development beyond clinical settings.

To conclude, advancing evidence‐based pediatric sleep practices is essential for meeting the evolving needs of children and families. To harness the long‐term benefits of sleep and develop effective evidence‐driven strategies, I have outlined several key action points. By prioritizing research, improving clinical training, and incorporating patient preferences, pediatric sleep management can evolve to address the unique demands of Generation Alpha. This transformation will ultimately lead to better health outcomes, ensuring that each child receives personalized, context‐specific care. Through a collaborative evidence‐driven approach to pediatric sleep practices, we can help nurture healthier generations in the years to come.

## 5 The Power of Pediatric Sleep Action Points: Prioritizing Pediatric Sleep Health

**Table:** 

Prioritizing pediatric sleep research	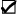 Support multidisciplinary research
	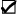 Empower cultural normativeness and beliefs
	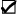 Prioritize sleep development in children
	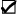 Develop and distribute evidence‐based pediatric sleep guidelines
	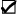 Leverage technology in sleep health initiatives
Improving clinical training	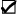 Integrate sleep health education into health care professionals' curricula
	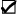 Train healthcare providers in sleep education, intervention programs, and cultural competence
	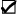 Expand sleep health training for non‐specialists (e.g., teachers)
Incorporating patient preferences	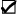 Engage youth and families in sleep education initiatives
	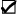 Encourage shared decision‐making in the management of sleep and sleep‐related disorders
	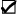 Integrate sleep health into child mental health initiatives, welfare, and education programs
	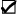 Co‐develop and implement digital tools for sleep health
Collaborative, evidence‐driven approach	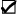 Regularly update and produce evidence‐based guidelines
	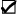 Establish pediatric sleep networks and consortia
	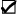 Foster global and local partnerships, collaborating with technology developers
	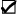 Design evidence‐driven strategies focused on long‐term outcomes
	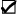 Implement large‐scale pediatric sleep data collection efforts
Integrating pediatric sleep into national health priorities	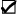 Prioritize pediatric sleep as a public health issue
	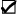 Align sleep health initiatives with broader health goals (e.g., physical activity, nutrition, and education)
	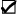 Advocate for preventive pediatric sleep health care services
	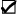 Secure funding pediatric sleep research
	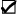 Ensure equitable access to pediatric sleep health care services and technology
	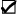 Ensure insurance coverage for sleep health programs
	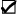 Incorporate sleep education into school curricula
	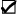 Launch nationwide public health campaigns focusing on sleep health
	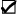 Advocate for policy changes that promote sleep‐friendly practices and healthy family sleep

## Author Contributions

The author takes full responsibility for this article.

## Conflicts of Interest

Prof. Karen Spruyt is deputy editor‐in‐chief of *Pediatric Discovery*. And she was excluded from all decision‐making related to the acceptance for publication. Apart from this, the author declares no conflicts of interest.

## Data Availability

The author has nothing to report.
